# Impact of being physically active on the brain electrocortical activity, brain volumetry and performance in the Stroop color and word test in women with fibromyalgia

**DOI:** 10.1038/s41598-022-16903-y

**Published:** 2022-07-23

**Authors:** Santos Villafaina, Juan Luis Leon-Llamas, Alvaro Murillo-Garcia, Narcis Gusi

**Affiliations:** 1 Universidad de Extremadura, Facultad de Ciencias del Deporte, Grupo de Investigación Actividad Física y Calidad de Vida (AFYCAV), Av. De Universidad s/n, 10003 Caceres, Spain; 2 Departamento de Desporto e Saúde, Escola de Saúde e Desenvolvimento Humano, Universidade de Évora, Évora, Portugal; 3 Biomedical Research Networking Center on Frailty and Healthy Aging (CIBERFES), Madrid, Spain

**Keywords:** Neuroscience, Rheumatic diseases

## Abstract

Physical exercise is one of the treatment approaches with the most robust evidence against fibromyalgia (FM) symptoms. This study aimed to investigate the impact of being physically active on the Stroop Color and Word Test (SCWT) performance as well as to investigate and compare the brain electrocortical activity during SCWT. A total of 31 women completed the SCWT while EEG was recorded. People with FM were divided into two groups (physically and non-physically active) according to the WHO guidelines. Furthermore, magnetic resonance imaging was acquired and health-related quality of life, the impact of the disease, and the six-minute walking test were administered. Physically active group showed better performance in the SCWT, exhibiting less error in name different color patches condition (C), more correct responses in named color-word condition (CW) and higher interference score than non-physically active group. Moreover, a significantly higher theta power spectrum in the Fp1 during the condition C in the SCWT and a higher volume in the right rostral middle frontal gyrus have been found in the physically active group. Furthermore, physically active women with FM showed positively correlations between correct responses in names of colors printed in black condition (W) in the SCWT and theta power in the F3, Fz, Fp2 and F4 scalp positions. Regarding non-physically active women with FM, errors in condition CW negatively correlated with the volume of left superior frontal gyrus, left rostral middle frontal gyrus, right rostral middle frontal gyrus, left caudal middle frontal gyrus and right caudal middle frontal gyrus. Furthermore, physically active group showed increased performance in the 6 min walking test and lower disease impact. Fulfil the physical activity recommendation seems to protect brain health since better SCWT performance, greater frontal theta power and higher volume in the right rostral middle frontal gyrus have been found in physically active women with FM.

## Introduction

Fibromyalgia (FM) is characterized by chronic, widespread, and persistent pain, which also can be accompanied by other symptoms like cognitive impairment, stiffness, depression, sleep disorders, anxiety or mobility impairments^1^. Its prevalence is around 2–3% worldwide^2^. Regarding cognition, impairments of executive functions, specifically selective attention, inference inhibition and processing speed, assessed by the Stroop Color and Word Test (SCWT) have been shown in people with FM^3–5^. In connection with this behavioral result, people with FM showed a reduced grey matter of numerous brain structures related to executive functions like anterior cingulate^6–8^, prefrontal cortex^6^, amygdala^6^, or hippocampus^9–11^. There is moderate evidence that the decrease in grey matter volume in this brain regions are related to pain processing (cingulate, insular and prefrontal cortices) and stress (mainly parahippocampal gyrus)^12^. Thus, it has been suggested that people with FM experience cognitive problems when these overlapping brain systems are preferentially engaged in processing painful stimuli^13^. In order to clarify the impact of these brain volume differences on executive function, a previous study conducted the Go/NoGo task while undergoing functional magnetic resonance imaging (fMRI). Authors found less activation in the premotor cortex, insular cortex and inferior frontal gyrus in people with FM than in healthy controls.

Despite volumetric differences in people with FM, previous studies have shown an abnormal EEG signal at rest^14–16^ compared to healthy controls, linked to hippocampus atrophy^11,15^. Regarding EEG abnormalities in people with FM, alterations to resting-state oscillatory activity in theta power spectrum were found^16^. These alterations were located in the frontal brain regions and may contribute to persistent pain in people with FM or represent the outcome of prolonged symptoms^16^. In the same line, a previous study also showed alterations of theta power spectrum in women with FM^15^. These alterations were correlated with the duration of symptoms^15^. In addition, people with FM also showed altered brain EEG activations during cognitive tasks^17–19^. In this regard, a reduced frontal brain activity during performance of an interference task was found^17^. This reduced brain activity was associated with the patients' cognitive complaints. Moreover, Samartin-Veiga, González-Villar and Carrillo-de-la-Peña^17^ also showed an abnormal P3 which suggested and altered modulation of attention according to the task demands in FM patients. González-Villar, Pidal-Miranda, Arias, Rodríguez-Salgado and Carrillo-de-la-Peña^18^ showed smaller power increase in midfrontal theta after stimulus presentation (2-back task) as well as reduced theta phase synchronization between midfrontal areas and other scalp electrodes in FM patients relative to healthy controls. In contrast, a previous study which investigated EEG activity while people with FM performed a reactive motor inhibition task, did not show significantly alteration related to inhibition (N2, P3, and midfrontal theta oscillations)^20^. However, this study found lower modulation of alpha which can suggest greater difficulty in mobilizing and maintaining visual attentional resources^20^.

Physical exercise is the treatment approach which presents strong evidence against the FM symptoms^21^. In this regard, previous studies have shown that physical exercise can improve health-related quality of life, pain or physical function^22–26^. In addition, changes in the EEG can be found 24-weeks of exergame intervention^27^. Authors showed an increase of beta-3 power spectrum in the frontal and temporal areas^27^. These changes can be associated with an increase cerebral blood flow promoted by physical exercise since beta power spectrum has been related to reoxygenation processes and hypoxia^28,29^. In the same line, Lardon and Polich^30^ showed that participants with a high level of fitness (enrolled in sports or with a minimum 3-year history of vigorous aerobic physical exercise) showed increased power in the theta, delta and beta bands at rest. Furthermore, active people with FM exhibited greater activity to painful stimuli in the left dorsolateral prefrontal cortex and posterior insula than non-physically active people with FM^31^. In the same line, Ellingson et al.^32^ reported that physical activity was positively related to brain responses during distraction form pain in dorsolateral prefrontal cortex, dorsal posterior cingulate and the periaqueducatal grey. In addition, sedentary time negatively related to areas involved in both pain modulation and the sensory-discriminative aspects of pain including the dorsolateral prefrontal cortex (DLPFC), thalamus and superior frontal and pre and postcentral gyri^32^. These findings are relevant since people with FM tend to be less physically active than healthy controls^33^, and therefore, showing a reduced physical fitness^34^.

Since attentional resources are limited, different stimuli compete with each other for attentional space. In this regard, previous studies have shown that people with FM showed lower dual-task performance (motor-cognitive) than healthy controls^35,36^. However, Martín‐Martínez et al.^37^ showed that 24-weeks of exergame intervention improved dual-task performance. In the same line, Martinsen, Flodin, Berrebi, Löfgren, Bileviciute‐Ljungar, Mannerkorpi, Ingvar, Fransson and Kosek^5^ reported a significant effect of a long-term exercise intervention on the speed of cognitive processing during SCWT in people with FM. In this study, authors showed improvements in the reaction times in people with FM after the intervention. Considering that people with FM exhibited greater reaction times when difficulty increased^38^, these results could indicate a normalization of executive function related to task difficulty after exercise interventions. Thus, physical exercise can be considered as an enhancer environmental factor promoting neuroplasticity^39^, promoting cellular (neurogenesis, synaptogenesis or angiogenesis), molecular (changes in neurotransmission systems or neurotrophic factors)^40^ and behavioral changes (improvements on behavioral tests)^41^.

Progress in wireless technologies allowed to study neurophysiological processes during ecological scenarios such as dual-task performance^35^ or electrocortical brain activity while eliciting depressive feelings^42^. To our knowledge, no previous studies have investigated the brain electrocortical activity during the SCWT in people with FM. In this regard, other studies have correlated the performance^43^ to cortical excitability or investigated brain activity using functional magnetic resonance imaging^5^. Furthermore, a previous study has investigated the effect of a long-term intervention on SCWT performance^5^. However, lower adherence to exercise has been reported in people with FM^44,45^. Thus, it would be interesting to investigate the impact of fulfilling the world health organization (WHO) guidelines on physical activity and sedentary behavior^46^ on the SCWT performance.

Therefore, the present study aimed: (1) to investigate the impact of being physically active (dividing the participants into two groups, physically and non-physically active, taking into account the WHO recommendations) on the SCWT performance (correct responses and errors in the three conditions of the SCWT test and interference score), (2) to investigate the differences between physically active and non-physically active women with FM on brain electrocortical activity (theta, alpha and beta power spectrums) during SCWT between two groups; (3) to investigate the differences between physically and non-physically active women with FM in brain volumetry (focused on caudate, insula, hippocampus and DLPFC). We hypothesized that physically active people with FM would have better SCWT performance, exhibited higher EEG theta power spectrum during SCWT in the frontal scalp positions, and greater volumes of brain areas related to SCWT performance (caudate, insula, hippocampus and DLPFC) than people who did not fulfil the WHO recommendations.

## Results

### Participant characteristics

Table 1 shows the participants’ characteristics. Physically active women with FM reported significant lower impact of the disease than non-physically active women with FM. Furthermore, physically active women with FM reported higher performance in the 6-minute walking test and significantly higher minutes of vigorous and moderate than non-physically active women with FM. Significant differences between groups were not observed in age, medication intake, cognitive impairments, or duration of FM symptoms.

**Table 1:** Participants’ characteristics.

Measurements	Physically active women with FM Mean (SD)	Non-physically women with FM Mean (SD)	p-value	Effect size	Contrast
Sample size (N)	16	15			
Age (years)	52.56 (8.09)	53.29 (7.39)	0.751	0.057	− 0.317
**Medication intake (%)**					
Antidepressant	6 (37.5%)	10 (66.67%)	0.104	0.085	2.635
Analgesics/Relaxants	4 (25%)	8 (53.33%)	0.106	0.084	2.620
Diuretics	1 (6.25%)	1 (6.67%)	0.926	< 0.001	0.002
Hypotensive	3 (18.75%)	1 (6.67%)	0.316	0.032	1.006
Others	7 (43.75)	12 (80%)	0.089	0.093	2.896
MMSE	33.94 (1.53)	33 (2.03)	0.133	0.270	− 1.502
**Educational level**					
Primary school (n)	7	5			
Secondary-school(n)	8	5	0.172	0.161	4.999
Degree	1	5			
BMI (kg/m^2^)	29.10 (4.58)	26.08 (3.46)	0.109	0.287	− 1.601
Fat mass (%)	29.21 (7.53)	24.54 (7.08)	0.101	0.294	− 1.641
Duration of FM symptoms (years)	20.75 (14.48)	19.25 (14.52)	0.545	0.108	− 0.605
FIQR	48.75 (18.84)	66 (17.24)	0.028	0.433	− 2.194
VAS for pain (0–100)	54.69 (19.96)	65.33 (15.52)	0.100	0.295	− 1.645
EQ-5D-5L	0.57 (0.23)	0.52 (0.25)	0.353	0.167	− 0.929
Six-minute walking test (m)	530.67 (39.60)	467.63 (74.60)	0.016	0.433	− 2.413
IPAQ Minutes of moderate activity per week	405.94 (544.69)	12 (24.84)	< 0.001	0.702	− 3.908
IPAQ Minutes of vigorous activity per week	153.12 (292.50)	16.67 (28.70)	0.019	0.408	− 2.273

*BMI* Body mass index, *EQ-5D-5L* Euroqol-5 Dimensions-5 Levels, *MMSE* Mini-mental State Examination, *FIQR* Fibromyalgia impact questionnaire, *VAS* Visual Analogue Scale.

### Differences in the SWCT performance

Table 2 shows the differences between physically and non-physically active women with FM in the SCWT performance. Mann–Whitney U test showed that physically active women with FM reported significantly less error in C condition (p-value = 0.023) and more correct responses in CW condition (p-value = 0.027) than non-physically active women with FM. Moreover, significantly higher IG was achieved by physically active women with FM (p-value = 0.036).

**Table 2:** Differences between physically and non-physically active women with FM in SCWT performance.

Measurements	Physically active women with FM Mean (SD)	Non-physically women with FM Mean (SD)	p-value	Effect size	Contrast
Correct answers in W condition	103.31 (8.75)	96.60 (14.07)	0.159	0.252	− 1.407
Errors in W condition	0.19 (0.54)	0.6 (0.98)	0.160	0.252	− 1.406
Correct answers in C condition	82.56 (9.79)	76.67 (13.10)	0.143	0.263	− 1.464
Errors in C condition	0.06 (0.25)	0.6 (0.83)	0.023	0.408	− 2.273
Correct answers in CW condition	49.37 (9.72)	38.20 (12.94)	0.027	0.398	− 2.219
Errors in CW condition	1.25 (1.23)	2.20 (3)	0.414	0.147	− 0.817
Interference score	3.59 (7.90)	− 4.40 (11.48)	0.036	0.376	− 2.095

*W* names of colors printed in black, *C* names different color patches, *CW* names color-word, where color-word are printed in an incongruous color ink (name the color of the ink instead of reading the word).

Interference score was calculated with the following formula: IG = CW − [(W × C)/(W + C)].

Regarding within group differences, Friedmann test showed a significant reduction in the correct responses among all the conditions in both physically (p-value < 0.01) and non-physically active groups (p-value < 0.01). Physically active women with FM, showed significantly more correct responses in the names of colors printed in black (W) than in the name different color patches (C) (p-value = 0.008) and the named color-word (CW) condition (p-value < 0.001). Moreover, physically active women with FM exhibited significantly more correct responses in the C condition than in the CW condition (p-value = 0.004). In the same line, non-physically active women with FM also showed significantly more correct responses in the W condition than in the C condition (p-value = 0.018) and the CW condition (p-value < 0.001). Moreover, non-physically active women with FM exhibited significantly more correct responses in the C condition than in the CW condition (p-value = 0.003).

In relation to errors, Friedmann test showed a significant increase in the errors among the conditions in physically active (p-value < 0.01) and non-physically active (p-value = 0.015) women with FM. Physically active women with FM, showed significantly more errors in the CW condition than in the W condition (p-value = 0.017) and in the C conditions (p-value = 0.024). In the same line, non-physically active women with FM also showed significantly more errors in the CW condition than in the W (p-value = 0.045) and in the C conditions (p-value = 0.036).

### Electrocortical brain activation during SCWT

**Figure 1:**
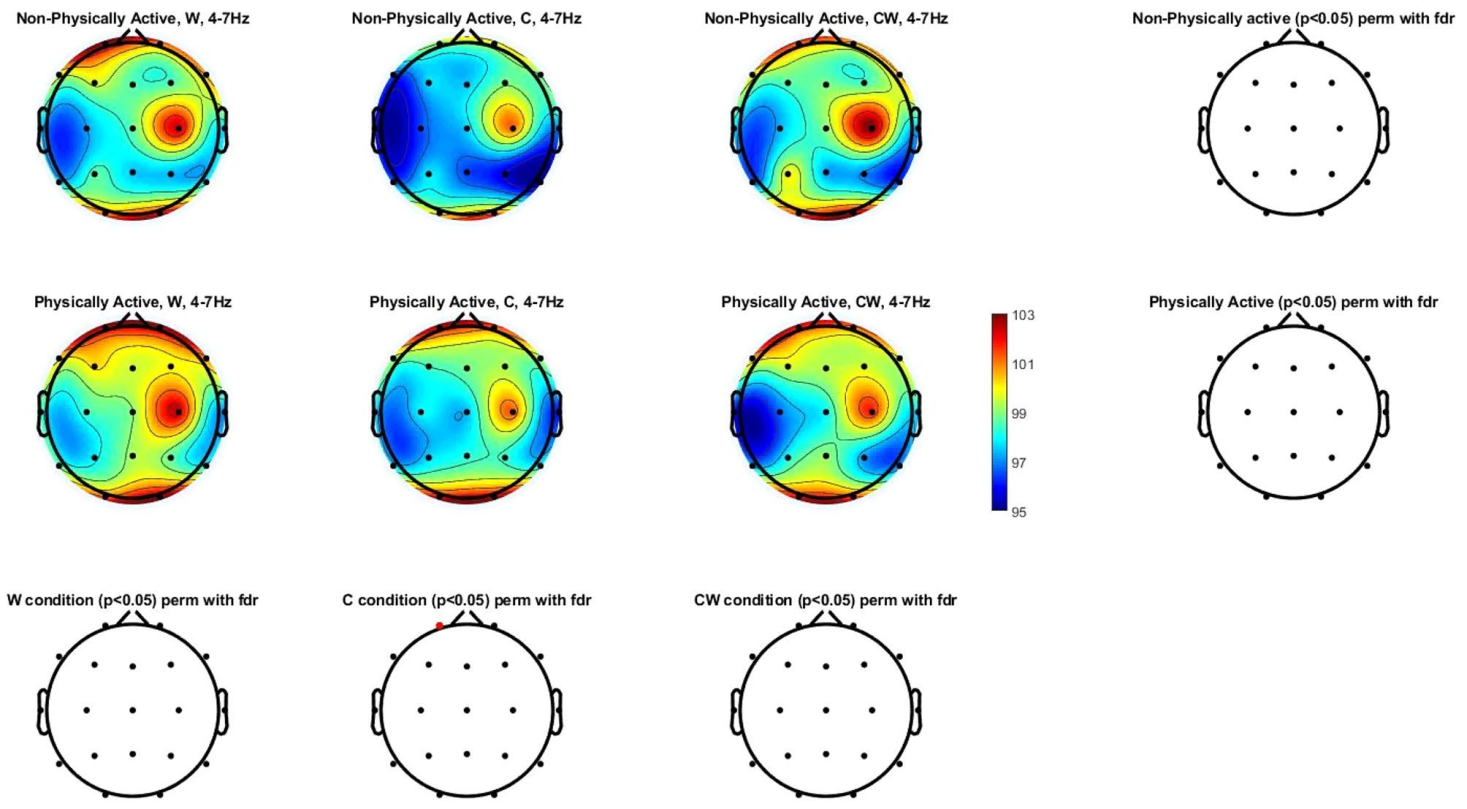
Theta power spectrum (4–7 Hz) topographic maps in physically and non-physically active women with FM. Differences were only located (p < 0.05, can be seen highlighted in red color) between physically and non-physically active women with FM in C condition in a frontal scalp location (Fp1). *W* names of colors printed in black, *C* names different color patches, *CW* names color-word, where color-word are printed in an incongruous color ink (name the color of the ink instead of reading the word).

Figure 1 shows the theta power spectrum (4–7 Hz) topographic maps in physically and non-physically active women with FM. Significant differences were only found in the Fp1 scalp location under the C condition (participants had to name different color patches). Differences in other scalp locations did not reach the significance level after applying FDR correction for multiple comparisons.

**Figure 2:**
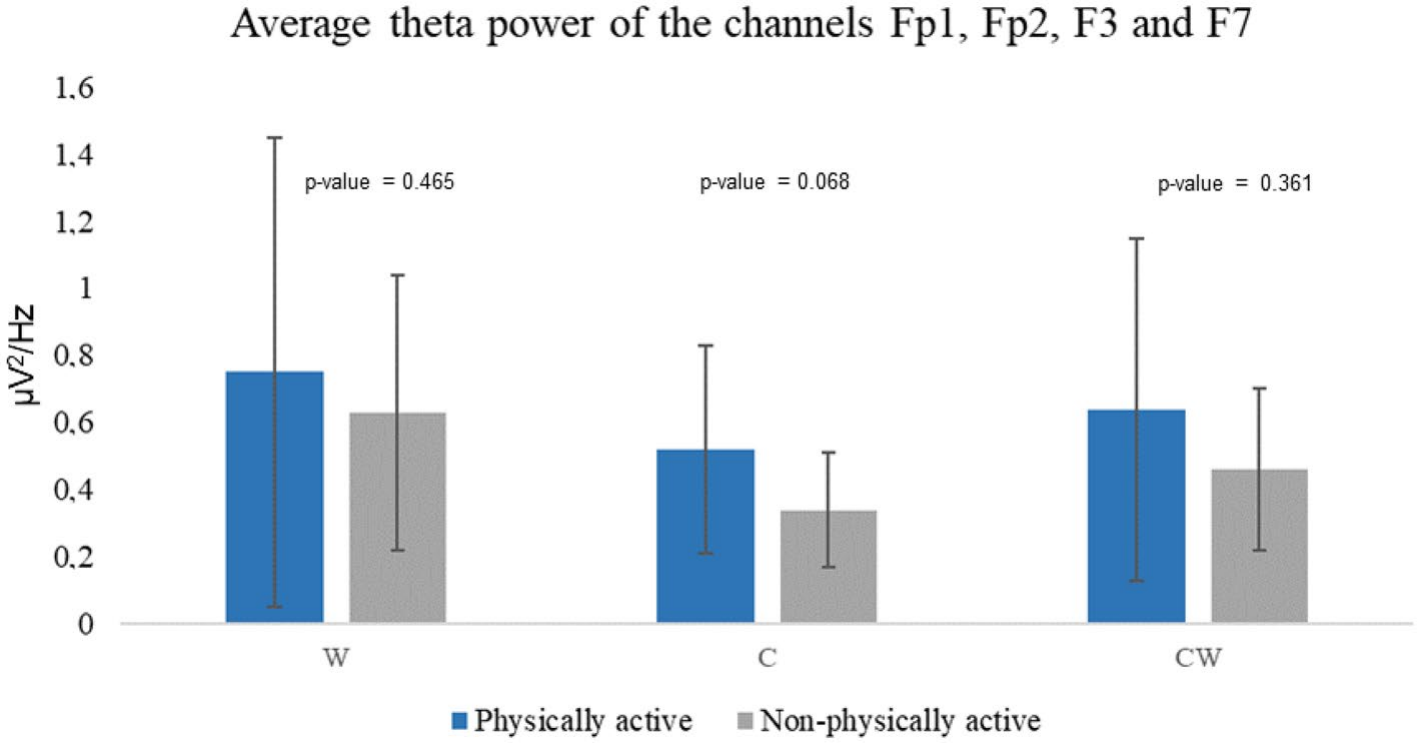
Average theta power spectrum (4–7 Hz) for Fp1, Fp2, F3 and F7 electrodes in physically and non-physically active women with FM. Differences were not found between or within groups. *W* names of colors printed in black, *C* names different color patches, *CW* names color-word, where color-word are printed in an incongruous color ink (name the color of the ink instead of reading the word).

Figure 2 shows the average theta power spectrum activity in the Fp1, Fp2, F3 and F7 electrodes. Between group comparison did not show significant differences. Moreover, Friedmann test did not show significant differences for physically active and non-physically active women (p-value = 0.127) with FM between conditions (p-value = 0.444).

**Figure 3:**
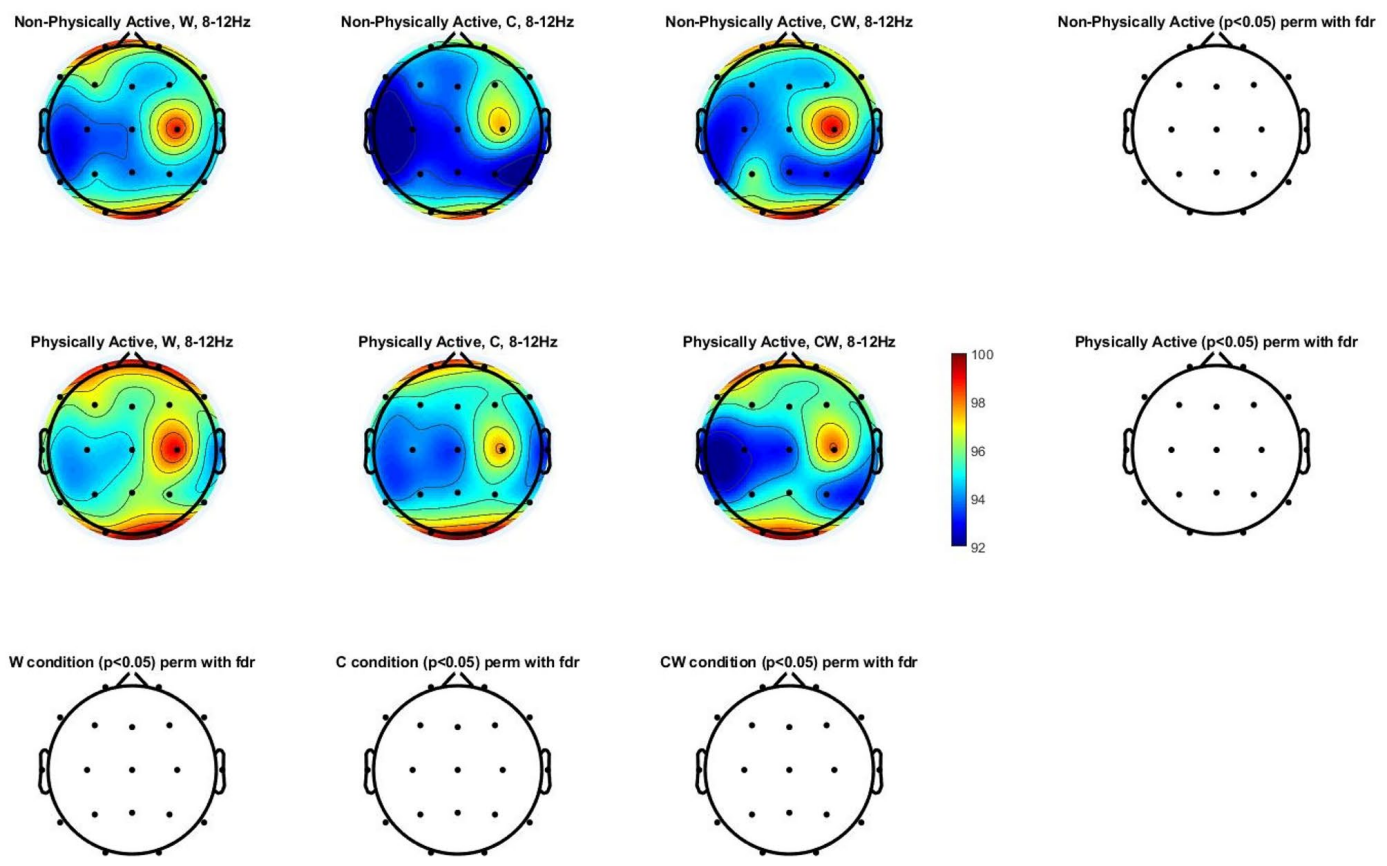
Alpha power spectrum (8–12 Hz) topographic maps in physically and non-physically active women with FM. Differences were not found (p > 0.05) in any of the scalp locations. *W* names of colors printed in black, *C* names different color patches, *CW* names color-word, where color-word are printed in an incongruous color ink (name the color of the ink instead of reading the word).

Figure 3 shows the alpha power spectrum (8–12 Hz) topographic maps in physically and non-physically active women with FM. Differences were not found in any of the scalp locations analyzed.

**Figure 4:**
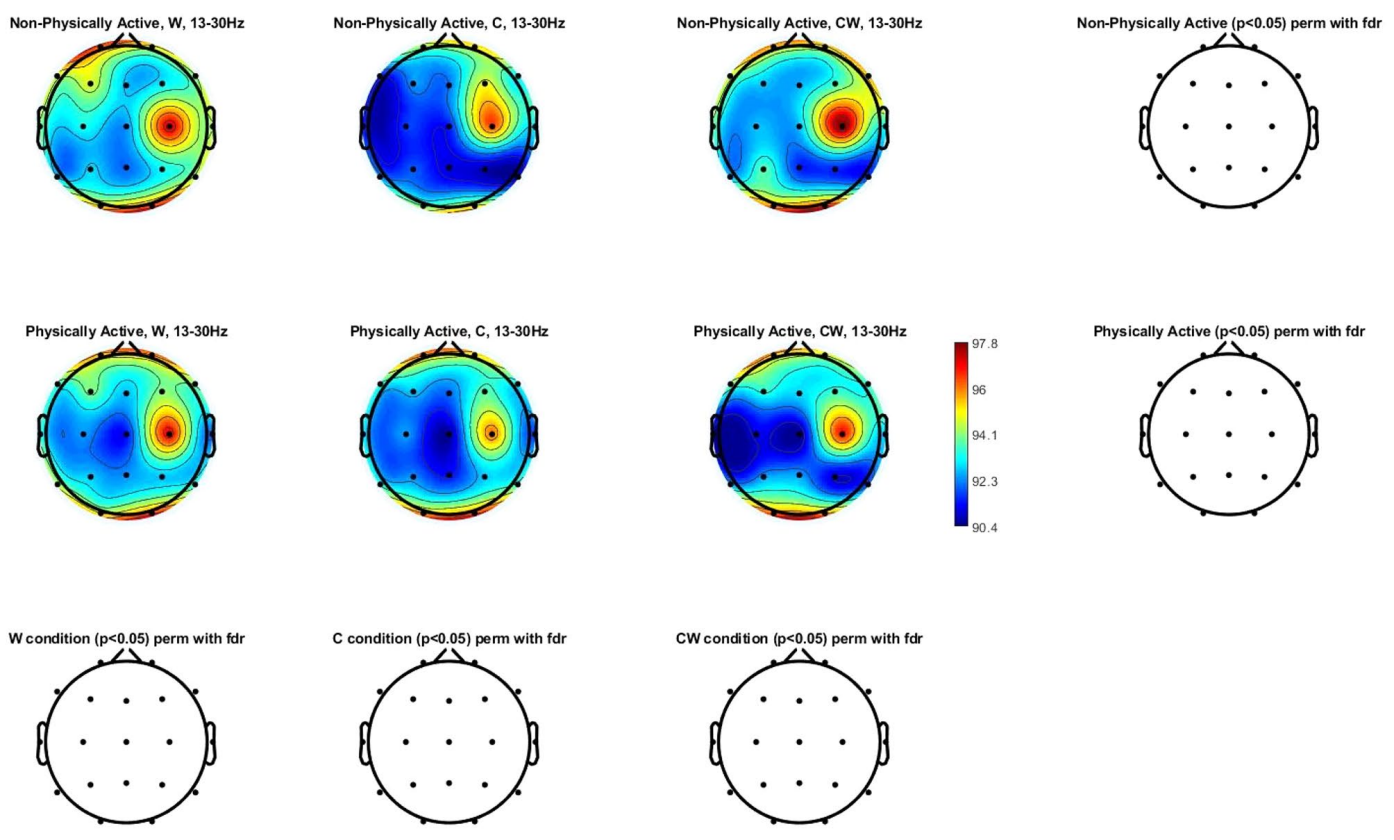
Beta power spectrum (13–30 Hz) topographic maps in physically and non-physically active women with FM. Differences were not found (p > 0.05) in any of the scalp locations. *W* names of colors printed in black, *C* names different color patches, *CW* names color-word, where color-word are printed in an incongruous color ink (name the color of the ink instead of reading the word).

Figure 4 shows the beta power spectrum (13–30 Hz) topographic maps in physically and non-physically active women with FM. Differences were not found in any of the scalp locations analyzed.

### MRI differences between physically and non-physically active women with FM

Table 3 shows the volumetric differences between physically and non-physically active women with FM in the amygdala, hippocampus, and dlPFC (superior frontal gyrus, rostral middle frontal gyrus, and caudal middle frontal gyrus). Significantly higher volumes in physically active women with FM were found in the right rostral middle frontal gyrus than in non-physically active women with FM.

**Table 3:** Volumetric differences between physically and non-physically active women with FM.

Measurements	Physically active women with FM Mean (SD) mm^3^	Non-physically women with FM Mean (SD) mm^3^	p-value	Effect size	Contrast
Left amygdala	1404.63 (185.74)	1346.93 (147.17)	0.125	0.306	− 1.533
Right amygdala	1660.81 (114.53)	1677.79 (182.76)	0.827	0.043	− 0.219
Left caudate	2617.56 (240.24)	2612.63 (498.97)	0.547	0.120	− 0.602
Right caudate	2790.61 (354.60)	2712.02 (436.68)	0.784	0.045	− 0.274
Left hippocampus	3985.63 (293.27)	3885.90 (389.30)	0.443	0.153	− 0.767
Right hippocampus	3982.22 (241.65)	3947.21 (412.77)	1.000	0	0
Left superior frontal gyrus	17,690 (2482.24)	16,157.6 (2909.49)	0.239	0.239	− 1.179
Right superior frontal gyrus	17,068 (2240.83)	16,115.81 (2302.31)	0.273	0.219	− 1.095
Left rostral middle frontal gyrus	12,443.31 (1588.09)	11,587 (2139.03)	0.137	0.298	− 1.489
Right rostral middle frontal gyrus	13,350.57 (2695.43)	11,802.81 (1612.89)	0.043	0.405	− 2.026
Left caudal middle frontal gyrus	4860.46 (928.65)	4312.4 (1039.86)	0.172	0.273	− 1.365
Right caudal middle frontal gyrus	4495.14 (1107.45)	4418.73 (804.28)	0.228	0.241	− 1.205

### Correlations between SCWT performance and MRI structures and frontal EEG theta power spectrum

Table 4 shows correlations between SCWT performance and magnetic resonance image (MRI) structures and frontal theta power spectrum in physically active women with FM. Significantly positive correlations were found between correct answers in W condition and theta power spectrum in F3 (ρ = 0.662, p-value = 0.011), Fz (ρ = 0.746, p-value = 0.007), Fp2 (ρ = 0.550, p-value = 0.047), and F4 (ρ = 0.723, p-value = 0.007) scalp locations. Regarding MRI structures, any of the studied structures were significantly correlated to SCWT performance in physically active women with FM.

**Table 4:** Correlations between SCWT performance and MRI structures and frontal EEG theta power spectrum in physically active women with fibromyalgia.

Variables	Correct answers in W condition	Errors in W condition	Correct answers in C condition	Errors in C condition	Correct answers in CW condition	Errors in CW condition	Interference score^a^	Interference score^b^	Interference score^c^
Left amygdala	0.040	0.103	0.088	0.103	− 0.013	0.374	− 0.262	–	–
Right amygdala	0.049	− 0.034	0.207	− 0.034	− 0.031	0.191	− 0.235	–	–
Left caudate	− 0.498	0.034	− 0.456	0.034	− 0.410	0.210	− 0.011	–	–
Right caudate	− 0.551	− 0.103	− 0.529	− 0.103	− 0.252	0.084	0.139	–	–
Left hippocampus	0.467	− 0.310	− 0.602	− 0.310	− 0.268	0.175	0.042	–	–
Right hippocampus	− 0.201	0.172	− 0.119	0.172	− 0.084	− 0.400	0.240	–	–
Left superior frontal gyrus	0.182	0.464	0.055	0.464	− 0.019	0.175	− 0.286	–	–
Right superior frontal gyrus	0.086	0.448	− 0.002	0.448	− 0.217	0.038	− 0.253	–	–
Left rostral middle frontal gyrus	− 0.257	0.464	− 0.050	0.464	0.113	0.019	0.006	–	–
Right rostral middle frontal gyrus	− 0.393	0.448	− 0.355	0.448	− 0.350	− 0.267	− 0.081	–	–
Left caudal middle frontal gyrus	− 0.212	0.386	− 0.264	0.386	− 0.608	0.155	− 0.545	–	–
Right caudal middle frontal gyrus	0.095	0.448	0.112	0.448	− 0.055	− 0.015	− 0.055	–	–
F7 electrode	0.273	0.077	0.251	0.140	− 0.433	0.150	− 0.156	− 0.259	− 0.371
F3 electrode	0.662***	0.330	0.015	0.420	− 0.122	0.092	− 0.356	− 0.041	− 0.291
Fp1 electrode	0.453	0.148	0.270	− 0.308	0.205	− 0.144	− 0.259	− 0.244	0.153
Fz electrode	0.746***	0.417	0.329	0.308	0.219	− 0.362	− 0.456	− 0.321	0.029
Fp2 electrode	0.550**	0.225	0.139	− 0.028	0.149	− 0.154	− 0.085	− 0.053	− 0.106
F4 electrode	0.732***	0.445	0.177	0.364	0.303	− 0.335	− 0.594	− 0.468	0.012
F8 electrode	0.223	0.143	0.161	0.420	0.049	− 0.206	− 0.135	− 0.276	− 0.062
Average of Fp2, Fp1, F3 and F7	0.482	0.187	0.285	− 0.028	− 0.196	− 0.010	− 0.265	− 0.247	− 0.074

*W* names of colors printed in black, *C* name different color patches, *CW* named color-word.

*p-value < 0.05; **p-value < 0.03; ***p-value < 0.01.

^a^The interference score was correlated with the EEG data recorded while participants performed the W condition.

^b^The interference score was correlated with the EEG data recorded while participants performed the C condition.

^c^The interference score was correlated with the EEG data recorded while participants performed the CW condition.

Table 5 shows the correlations between SCWT performance and MRI structures and frontal theta power spectrum in non-physically active women with FM. Regarding MRI structures, errors in CW condition negatively correlated with left superior frontal gyrus (ρ = 0.741, p-value = 0.021), left rostral middle frontal gyrus (ρ = 0.772, p-value = 0.020), right rostral middle frontal gyrus (ρ = 0.770, p-value = 0.020), left caudal middle frontal gyrus (ρ = 0.667, p-value = 0.042), and right caudal middle frontal gyrus (ρ = 0.738, p-value = 0.020). Furthermore, neurophysiological variables showed that Fz theta power spectrum negatively correlated with errors in C condition (ρ = − 0.683, p-value = 0.035).

**Table 5:** Correlations between SCWT performance and MRI structures and frontal EEG theta power spectrum in non-physically active women with fibromyalgia.

Variables	Correct answers in W condition	Errors in W condition	Correct answers in C condition	Errors in C condition	Correct answers in CW condition	Errors in CW condition	Interference score^a^	Interference score^b^	Interference score^c^
Left amygdala	0.105	0.375	− 0.245	0.146	− 0.437	− 0.297	− 0.373	–	–
Right amygdala	0.228	0.576	− 0.191	− 0.078	− 0.405	− 0.320	− 0.282	–	–
Left caudate	− 0.128	0.401	− 0.527	− 0.010	− 0.364	− 0.562	− 0.064	–	–
Right caudate	− 0.232	0.269	− 0.573	− 0.107	− 0.310	− 0.710	− 0.082	–	–
Left hippocampus	0.538	0.338	0.018	− 0.078	− 0.164	− 0.330	− 0.182	–	–
Right hippocampus	0.620	0.417	0.218	− 0.360	0.009	− 0.241	− 0.082	–	–
Left superior frontal gyrus	− 0.122	− 0.037	− 0.467	− 0.066	− 0.012	− 0.741**	− 0.006	–	–
Right superior frontal gyrus	0.109	0.048	− 0.100	0.438	− 0.396	− 0.339	− 0.364	–	–
Left rostral middle frontal gyrus	− 0.201	− 0.425	− 0.345	0.007	− 0.018	− 0.772***	− 0.152	–	–
Right rostral middle frontal gyrus	− 0.068	− 0.201	− 0.218	0.039	− 0.191	− 0.770***	− 0.227	–	–
Left caudal middle frontal gyrus	− 0.383	− 0.619	− 0.527	0.211	− 0.061	− 0.667*	− 0.127	–	–
Right caudal middle frontal gyrus	− 0.100	− 0.560	− 0.391	− 0.049	0.082	− 0.738**	− 0.018	–	–
F7 electrode	0.242	0.379	0.379	0.134	0.059	0.205	− 0.254	0.314	− 0.168
F3 electrode	0.181	0.066	0.039	− 0.573	0.057	0.274	0.079	− 0.079	− 0.232
Fp1 electrode	0.079	0.168	− 0.218	0.134	− 0.266	0.219	0.039	0.086	− 0.471
Fz electrode	0.011	0.495	0.081	− 0.683***	− 0.197	0.177	0.104	− 0.125	− 0.364
Fp2 electrode	0.295	0.220	− 0.055	0.281	− 0.016	0.022	0.307	0.096	− 0.218
F4 electrode	0.107	0.512	− 0.098	0.098	− 0.436	0.033	0.029	− 0.032	− 0.436
F8 electrode	0.576	0.194	0.016	0.195	0.365	− 0.245	0.071	− 0.129	0.418
Average of Fp2, Fp1, F3 and F7	0.240	0.196	0.025	0.085	0.005	0.256	0.064	0.025	− 0.275

*W* names of colors printed in black, *C* name different color patches, *CW* named color-word.

*p-value < 0.05; **p-value < 0.03; ***p-value < 0.01.

^a^The interference score was correlated with the EEG data recorded while participants performed the W condition.

^b^The interference score was correlated with the EEG data recorded while participants performed the C condition.

^c^The interference score was correlated with the EEG data recorded while participants performed the CW condition.

## Discussion

This study aimed to explore the impact of being physically active in the SCWT performance, and electrocortical brain activation during SCWT in women with FM. Furthermore, this study also aimed to explore the volumetric differences between physically and non-physically active women with FM in brain structures involved during SCWT processing using MRI. Results showed that both groups significantly differed in the impact of the disease and performance in the 6-min walking test, with worst results corresponding to non-physically active women with FM. Moreover, differences between physically and non-physically active women with FM can be found in SWCT performance in C and CW condition, as well in the IG score. Regarding EEG results, differences between groups can be found in the theta power spectrum, specifically in the Fp1 scalp location during the C condition and significantly correlations were found between theta power spectrum in the frontal area (located in the F3, Fz, Fp2 and F4 scalp positions) and correct answers in W condition in physically active women with FM. Furthermore, between groups differences can be observed in the MRI, with higher volumes of the right rostral middle frontal gyrus observed in the physically active women with FM. In non-physically active women with FM, errors in CW condition negatively correlated with left superior frontal gyrus, left rostral middle frontal gyrus, right rostral middle frontal gyrus, left caudal middle frontal gyrus, and right caudal middle frontal gyrus volumes.

Previous studies have highlighted the importance of the physical activity to maintain and improve different cognitive domains such as spatial memory, working memory, and executive attention^47,48^. This is relevant since people with FM have shown cognitive impairments related to cognitive flexibility^4^, decision making^49^, memory^50^ or processing speed^51^. In this regard, previous studies showed that a long-term physical activity intervention could normalize the executive function related to task difficulty, that is reduced inference inhibition^5,38^ measured by the SCWT. Improvements or normalization of SCWT performance due to exercise could be in line with our results, where physically active women with FM showed higher performance in the C condition (reporting fewer errors), in the CW condition (reporting more correct responses) as well as higher IG than non-physically active women with FM. Regarding IG, in our study non-physically active women with FM had a pathological ability to inhibit interference (since values are negative)^52^, whereas physically active women with FM did not. Previous studies showed that exercise could increase angiogenesis^53,54^ and concentrations of brain-derived neurotrophic factor (BDNF), which is involved in different aspects such as neuroplasticity, neurogenesis, synaptogenesis, or cognition, among others^55,56^. Furthermore, this neurotrophic factor is a mediator of executive function^57^. Thus, we hypothesized that the observed difference between physically and non-physically active women with FM might be due to the protective effect of exercise on brain health^55^.

In this regard, physically active women with FM showed significantly higher performance in the 6-min walking test as well as lower values in the impact of the diseases. In addition, although not significantly, physically active women with FM showed higher health-related quality of life or lower pain level. This is relevant since these results could evidence the vicious circle of people with FM in which pain forces patients to stop certain activities, and the consequence of this is even more pain and, therefore, lower health-related quality of life. Thus, this vicious circle is aggravated by physical inactivity^58^. This article shows that this vicious circle can be broken by physical activity as a previous article suggested^59^. Furthermore, once this vicious circle has been broken increments in cognitive function, physical fitness or impact of the disease can be observed. However, in the present study we were unable to discern whether participants in the physically active group have lower disease impact of their physical activity, or are able to be physically active because they have lower impact. Therefore, results must be taken with caution.

Previous studies have analyzed the brain structures involved during SCWT. In this regard, the activation in the dlPFC is related to a positive performance in this test^60^. Furthermore, a previous study focused on people with FM has also shown a reduced activation in the caudate nucleus, hippocampus, and amygdala in people with FM during the SCWT^5,38^. Martinsen, Flodin, Berrebi, Löfgren, Bileviciute‐Ljungar, Mannerkorpi, Ingvar, Fransson and Kosek^5^ investigated if a long-term physical activity intervention could modify brain activation, measured by fMRI, during SCWT. Thus, we decided to explore if SCWT brain-related structures (caudate, hippocampus, amygdala, and dlPFC) of physically and non-physically women with FM significantly differed. Results showed that significant differences could be found in the right rostral middle frontal gyrus. This is relevant since the right middle frontal gyrus is active only when reorienting to unexpected stimuli^61,62^. Furthermore, in a previous study, this structure was proposed as the link between ventral and dorsal networks, reorienting the person’s attention to a novel task or a relevant external stimulus^63,64^. Therefore, considering the functions of the right middle frontal gyrus, this structure could significantly impact SCWT performance due to the attentional requirements of this test^65^. Furthermore, our results also showed that, in non-physically active women with FM, errors in CW condition negatively correlated with left superior frontal gyrus, left rostral middle frontal gyrus, right rostral middle frontal gyrus, left caudal middle frontal gyrus, and right caudal middle frontal gyrus volumes.

Previous studies have found that people with FM have abnormal electrocortical brain activity measured by EEG power spectrum^14–16^. Even the years they have been suffering from FM seem to affect the EEG power spectrum^15^. In this regard, a previous study has shown that six months of physical activity intervention could modify the EEG beta-3 power spectrum^27^. The authors pointed out that these modifications could be due to increments in the cerebral blood flow^27^. Our results showed that during the C condition, higher values of theta power spectrum are achieved by physically active women with FM in the Fp1 electrode. The area measured by this electrode corresponds morphologically to the most rostral part of the left superior frontal gyrus, which is part of the DLPFC, a structure that was shown to be involved during SCWT^5,38,60^. Furthermore, physical active women with FM showed positive correlation between theta power spectrum in the frontal area (located in the F3, Fz, Fp2 and F4 scalp positions) and correct answers in W condition. In contrast, non-physically active women with FM showed a negative correlation between theta power spectrum at Fz and errors in C condition. Regarding theta power spectrum, previous studies indicated that when task difficulty increases or higher level of mental effort is required, the power spectrum is increased^66–68^. Thus, frontal theta has been considered a potential indicator of cognitive effort or success^69^. In people with FM previous studies have found abnormalities in this frequency spectrum band at rest^16^ and during cognitive task or stimulus^17–19^. For instance, people with FM previous study showed smaller power increase in midfrontal theta after stimulus presentation (2-back task) than healthy controls^18^. Probably, our results can be explained by the limited resource theory^70^ since processing pain takes over the resources needed for cognition. Taking all this information together, theta power spectrum results and limited resource theory could explain why a lower performance in the C condition (more errors) can be found in non-physically active women with FM. However, these differences could be even higher in working memory tasks where prefrontal areas are also considered as key structures. Thus, future studies should investigate the neurophysiological response of physically and non-physically active women with FM during working memory task.

This study has some limitations that should be acknowledged. First, the sample was only comprised of women, so future researchers should expand the study to include men and participants of different ages. Second, the relatively small sample size and the p-value adjustment for multiple comparisons could have made that only greater differences have reached the significance level. Third, differences obtained in the impact of the disease could hypothetically be playing a role in the differences found in SCWT performance, cortical EEG activity or even brain volumetry. Thus, future longitudinal studies or randomized controlled trials are encouraged to isolate the impact of physical activity on brain volumetry, cortical EEG activity, and SCWT performance. Fourth, in the present study the MMSE was used as a screening tool for cognitive impairment. However, a recent study suggested that MMSE could show ceiling effect in women with FM^71^ and another test such as the Montreal Cognitive Assessment (MoCA) test should be used. Thus, future studies in people with FM should incorporated this test instead of MMSE to characterize the cognitive impairment in this population.

In conclusion, our study found that physically active women with FM showed better performance in the SCWT than non-physically active women with FM. Furthermore, a significantly higher theta power spectrum during the SCWT and a higher volume in the right rostral middle frontal gyrus have been found in the physically active women with FM and could explain the higher performance in this test. Furthermore, physically active women with FM showed increased performance in the 6 min walking test as well as lower disease impact. Therefore, fulfilling the physical activity recommendation (150–300 min of moderate-intensity, or 75–150 min of vigorous-intensity physical activity) seems to protect the brain health of women with FM.

## Methods

### Participants

A total of 31 women (age 52.87 (7.64)) participated in this cross-sectional study. People with FM were recruited by telephone calls by the Association of Fibromyalgia (AFIBROEX). Inclusion criteria were: (a) be diagnosed according to the American College of Rheumatology's criteria^72^, (b) be a female and aged between 30 and 65 years, (c) be able to communicate with the research staff, (d) have read and signed the written informed consent. Participants were excluded if they: (a) had contraindications for physical exercise, (b) suffered from a psychiatric disorder which can lead to cognitive impairment, a neurological disorder or brain injury, and (c) were pregnant. Procedures were approved by the University bioethical committee (approval number: 62/2017), in accordance with the updated Declaration of Helsinki. All the participants read and signed the informed consent prior to the first assessment.

According to the WHO recommendation guidelines on physical activity and sedentary behavior^46^, adult people should undertake 150–300 min of moderate-intensity, or 75–150 min of vigorous-intensity physical activity, or some equivalent combination of moderate-intensity and vigorous-intensity aerobic physical activity, per week. Therefore, we divided our sample size between physically active (age 52.56 (8.09)) and non-physically active people with FM (age 53.29 (7.39)). Characteristics and details of groups can be found in Table 1.

### Evaluations and questionnaires

The SCWT assesses the ability to inhibit cognitive interference, which occurs when the processing of a stimulus feature affects the simultaneous processing of another attribute of the same stimulus^73^. In this test, participants were asked to read three different tables (two congruous conditions and one incongruous condition) as fast as possible: (1) names of colors printed in black (W); (2) name different color patches (C); and (3) named color-word (CW) where color-word are printed in an incongruous color ink (i.e., the word green is printed in blue ink). In this incongruous condition, participants are asked to name the color of the ink instead of reading the word. Numbers of errors and correct responses were registered during the 45 s, which lasted each condition. Furthermore, the following formula was calculated in order to extract the interference score (IG)^52,74^:

$$IG = CW \, {-} \, \left[ {\left( {W \, \times \, C} \right)/\left( {W + \, C} \right)} \right],$$

where IG is the Interference score; CW is the number of correct responses in 45 s in the CW condition; W is the number of correct responses in 45 s in the W condition; C is the number of correct responses in 45 s in the C condition.

The health-related quality of life was assessed using the Spanish version of the Euroqol-5 Dimensions-5 Levels (EQ-5D-5L)^75^. This questionnaire comprises five dimensions (mobility, self-care, daily life activities, pain or discomfort, and anxiety or depression) and five levels per dimension. The EQ-5D-5L utility index evaluates the perceived health status from 0 (the worst health-related quality of life) to 1 (the best health-related quality of life).

The pain level was evaluated through a VAS for pain (0–100), asking by the intensity of pain referring to the day they were evaluated.

The impact of the disease was assessed using the Spanish version of the Fibromyalgia Impact Questionnaire Revised (FIQ-R)^76^. The FIQ-R^77^ has 21 items scored from 0 to 10, representing 10 as the worst condition. It is divided into three domains: (a) function, (b) overall impact, and (c) symptoms. The maximum score is 100, which corresponds to the worst overall symptom impact.

The Mini-Mental State Examination (MMSE) is a widely used screening tool for dementia^78^ and mild cognitive impairment^79^, and it is validated for Spanish-speaking communities^80^. Values under 23/24 are considered cognitive impairment^79^.

The 6-min walking test was used to assess the cardiorespiratory fitness of women with FM. The distance, in meters, that participants could walk during the 6 min was assessed^81,82^.

In order to evaluate physical activity habits, the International Physical Activity Questionnaire (IPAQ) was used^83^. The nine items of the questionnaire provided information on the time spent walking, in vigorous- and moderate intensity activity, and in sedentary activity. This questionnaire was used to assess if participants fulfilled the WHO recommendation guidelines on physical activity and sedentary behavior^46^.

In addition, participants were heighted and weighted using a stadiometer (SECA 225, SECA, Hamburg, Germany) in order to calculate the body mass index (BMI), and body composition was assessed using a Tanita Body Composition Analyzer (TANITA BC-418MA). Age, durations of FM symptoms, educational level and medication intake were asked.

### EEG instrument and data processing

A total of 19 EEG scalp locations, according to the International 10–20 system, were recorded using an Enobio device (Neuroelectrics, Cambridge, MA, USA)^84^. These scalp locations were distributed as follows: frontal (Fz, Fp1, Fp2, F3, F4, F7, and F8), central (Cz, C3, and C4), temporal (T3, T4, T5, and T6), parietal (Pz, P3, and P4) and occipital (O1 and O2).

Electrodes placed in the earlobe were used as references, and impedance was kept below 10 kΩ during the recording. A sampling rate of 500 Hz was used. In order to perform the EEG pre-processing and data analysis, the EEGlab toolbox (MatLab) was used^85^. In this regard, the line noise was removed using a 1-Hz high-pass filter, and the Artifact Subspace Reconstruction (ASR) employed to reject bad channels and correct continuous data. Then, bad channels were interpolated, and data was re-references to average. Independent Component Analysis (ICA) was performed^86^, and single equivalent current dipoles estimated. The symmetrically constrained bilateral dipoles were searched. Independent Components (ICs) whose dipoles' residual variance is larger than 15% were removed as well as those with dipoles located outside the brain. Since neural oscillations are a fundamental property of the brain^87^, the time–frequency analysis provides direct information regarding the neurophysiological mechanism underlying the cognitive task administered^88^. Moreover, it is an extended methodology which allow comparison with previous studies. Thus, Power Spectral Density was computed and banded into theta (4–7 Hz), alpha (8–12) and beta (13–30) frequency bands.

### MRI instrument and data processing

T1-weighted images were acquired from a 3.0 Tesla scanner (Achieva 3.0 T TX, Philips Medical Systems, Best, Netherlands) with an 8-channel receiver head coil, using a 3D T1-weighted Turbo Field Echo. The parameters were set as follows: repetition time (TR) of 11.51 ms; echo time (TE) of 2.8 ms; 288 × 288 matrix size; 0.9 mm slice thickness; 10° flip angle; 1 number of averages.

All women were processed using FreeSurfer software 6.0 version (Laboratory for Computational Neuroimaging, Athinoula A. Martinos Center for Biomedical Imaging, Charlestown, MA, USA; http://surfer.nmr.mgh.harvard.edu) employing the “recon-all” pipeline on a MacBook Pro (Version OS X 10.14, 8 GB, 2.30 GHz, Intel Core i5). This software follows a well-described series of steps^89–92^, among which are the following: head motion correction and averaging; removal of non-brain tissue; automated Talairach space transformation; intensity normalization; segmentation of the subcortical and cortical structures using a probabilistic brain atlas; surface tessellation; topology correction; and surface deformation.

Hippocampus, amygdala, caudate and dorsolateral prefrontal cortex (dlPFC) were selected as brain structures involved during SCWT^5^. The dlPFC was composed of the superior frontal gyrus, rostral middle frontal gyrus, and caudal middle frontal gyrus^93^.

### Procedures

One researcher, which was not involved in the statistical analyses, evaluated all the participants. This researcher administered the EQ-5D-5L, the VAS for pain (0–100), the FIQ-R, the IPAQ and the MMSE as well as asked for their age, medication intake and the duration of the FM symptoms (years). Then, participants were weighted and heighted in order to obtain the body mass index (BMI) and the % Fat mass using the Tanita Body Composition Analyzer.

After questionnaires and body composition data were acquired, the EEG was placed and SCWT administered. The researcher and the participant were seated next to each other with an A4 sheet for each of the SCWT conditions with 100 possible responses distributed in columns. The SCWT consisted of three conditions, and following the SCWT guideline were administrated in the following order: (1) condition W: names of colors printed in black; condition C: name different color patches; and condition CW: named color-word where color-word are printed in an incongruous color ink. Each condition lasted 45 s, and they were encouraged to name as many words as possible (if the participant finished naming all the possible answers on the sheet, they started again). The EEG was recorded while participants performed the three conditions. The researcher assessed the performance (error and correct responses).

Once the three SCWT conditions were performed, participants conducted the 6-min walking test so that the fatigue caused by this test would not affect cognitive performance. After performing all these tests, the researcher provided an appointment to the MRI scan in the same week the procedure was conducted.

### Statistical analysis

The SPSS statistical package (version 22.0; SPSS, Inc., Chicago, Ill.) was used to analyze the health-related quality of life, impact of the disease, cardiorespiratory fitness, level of pain, brain structures, and SCWT. According to Shapiro–Wilk tests, non-parametric analyses were performed. Differences between groups were explored using Mann–Whitney U test. Within group differences were investigated using Friedman test as well as Wilcoxon signed rank test to conduct pairwise comparisons. Chi-Squared tests were conducted to explore differences between groups in the medication intake and educational level of participants. sizes, r (for Mann–Whitney U tests and Wilcoxon Signed Rank tests) and Kendall W (for Chi-Squared and Friedman test), were calculated^94^. Values of 0.37, 0.24, and 0.10 represent large, medium, and small effect sizes, respectively^95^. EEGLAB study design was used to compare the electrophysiological response during the SCWT in all the conditions (W, C and CW) both physically and non-physically active women with FM. Thus, an EEGLAB STUDY.design (2 × 3) was configured to compare women with FM with physically and non-physically active women with FM during three conditions. Non-parametric analysis (permutation analysis) was computed. In order to control the Type I error, the false discovery rate correction (FDR) was applied. Additionally, if differences were found at any one electrode, the power of the four neighboring electrodes (including itself) would be averaged and analyzed using the Mann–Whitney U test for between-group comparisons and the Friedman test for within-group comparisons. Furthermore, Spearman’s Rho correlations analyses were performed to analyze the relationship between SCWT performance and MRI and EEG data. Correlations were focused on MRI structures (hippocampus, amygdala, caudate and dlPFC structures) and frontal scalp locations (F7, F3, Fp1, Fz, Fp2, F4 and F8 electrodes) at theta power spectrum due to the differences obtained in the previous analyses. To decrease the probability of a Type I error, p-values have been corrected by Benjamini–Hochberg method^96^.

### Ethical standards

The authors assert that all procedures contributing to this work comply with the ethical standards of the relevant national and institutional committees on human experimentation and with the Helsinki Declaration of 1975, as revised in 2008.

## Data Availability

The datasets generated and/or analysed during the current study are not publicly available due that all participants gave their consent for the information to be kept confidential. However, it is possible to obtain the dataset from the corresponding author on reasonable request.
